# The Oncoprotein BCL11A Binds to Orphan Nuclear Receptor TLX and Potentiates its Transrepressive Function

**DOI:** 10.1371/journal.pone.0037963

**Published:** 2012-06-04

**Authors:** Sara B. Estruch, Víctor Buzón, Laia R. Carbó, Lenka Schorova, Jens Lüders, Eva Estébanez-Perpiñá

**Affiliations:** 1 Department of Biochemistry and Molecular Biology and Institute of Biomedicine from the University of Barcelona, University of Barcelona, Barcelona, Spain; 2 Cell and Developmental Biology Programme, Institute for Research in Biomedicine, Barcelona, Spain; University of South Florida, United States of America

## Abstract

Nuclear orphan receptor TLX (NR2E1) functions primarily as a transcriptional repressor and its pivotal role in brain development, glioblastoma, mental retardation and retinopathologies make it an attractive drug target. TLX is expressed in the neural stem cells (NSCs) of the subventricular zone and the hippocampus subgranular zone, regions with persistent neurogenesis in the adult brain, and functions as an essential regulator of NSCs maintenance and self-renewal. Little is known about the TLX social network of interactors and only few TLX coregulators are described. To identify and characterize novel TLX-binders and possible coregulators, we performed yeast-two-hybrid (Y2H) screens of a human adult brain cDNA library using different TLX constructs as baits. Our screens identified multiple clones of Atrophin-1 (ATN1), a previously described TLX interactor. In addition, we identified an interaction with the oncoprotein and zinc finger transcription factor BCL11A (CTIP1/Evi9), a key player in the hematopoietic system and in major blood-related malignancies. This interaction was validated by expression and coimmunoprecipitation in human cells. BCL11A potentiated the transrepressive function of TLX in an *in vitro* reporter gene assay. Our work suggests that BCL11A is a novel TLX coregulator that might be involved in TLX-dependent gene regulation in the brain.

## Introduction

TLX (NR2E1) is a transcription factor of the nuclear receptor superfamily (NRs), which was initially identified as the human homologue of the *Drosophila* tailless protein [1]. As in other members of the NR superfamily, structure-based sequence alignments and secondary structure predictions indicate that TLX features a DNA-binding domain (DBD), followed by a hinge region and a ligand binding domain (LBD) [2]. Whereas the DBD domain is implicated in binding to specific DNA sequences on target genes, the LBD domain engages in macromolecular complexes that ultimately regulate gene transcription. The LBD domain is also the ligand-sensor domain in NRs whose ligands have been identified. Whether this is the case for TLX remains to be experimentally determined, hence this receptor is classified as an orphan NR.

TLX plays major roles both in brain functions and behavior. Adult mice lacking TLX present smaller brains than their wild-type littermates with reduced cerebral hemispheres, olfactory bulbs and hippocampus, as well as thinning of the neocortex [3]–[10]. TLX-null mice also show severe behavioral and emotional alterations such as aggressivity, hyperactivity and learning disabilities [11]–[13]. TLX seems to be only expressed in the subventricular zone of the lateral ventricles and the subgranular zone in the hippocampus dentate gyrus, which are well-established neurogenesis regions in the adult brain [6] [9]–[10] [13]. Shi *et al.* demonstrated that the adult neural stem cell pool (NSCs) of the neurogenic brain areas is composed of TLX-positive cells, which can self-renew and generate differentiated cells in the nervous system [6]. TLX is a key regulator of the timing of neurogenesis in the cortex and NSCs self-renewal in adult brains [6]–[7] [14]–[15]. TLX also plays a role in eye development and vision, as well as in retinopathies and eye malformations [16]–[17].

TLX has been proposed to function primarily as a transcriptional repressor of target genes through its physical interactions with transcriptional corepressors including epigenetic modifiers like lysine-specific histone demethylase 1 (LSD1) [18]–[20]. TLX also binds to atrophin-1 (ATN1), which belongs to a newly identified class of NR corepressors [16] [21]–[25]. The direct association between TLX and ATN1 prevents retinal dystrophy and TLX-null mice develop visual impairment [16]. ATN1 is involved in the human neurodegeneration called dentatorubral-pallidoluysian atrophy [25]–[26].

To identify and characterize novel protein interactors of human TLX in the adult brain and possible coregulators of its function we performed yeast-two-hybrid (Y2H) screens of an adult brain cDNA library using full-length TLX (FL-TLX) and TLX-LBD constructs as baits. In our assays we identified several overlapping clones of human ATN1, thus confirming its physical association with TLX [16]. In addition, we describe that the oncoprotein and transcription factor B-cell lymphoma/leukemia 11A/CTIP1 (BCL11A) [27]–[28] is a novel interactor and regulator of TLX.

## Results

### TLX Interacts with Oncoprotein Bcl11a

To identify interactors of human TLX we generated human FL-TLX (1–385) and TLX-LBD (172–385) constructs and used these as baits to screen a Y2H human adult male brain library. Additionally, we also cloned the following TLX constructs to validate all identified clones in one-to-one Y2H assays: TLX LBD including the complete predicted hinge region (TLX-H-LBD: 94–385) and TLX-DBD flanked by the predicted N-terminal extension (TLX-NT-DBD: 1–95).

Human TLX domain boundaries are not strictly determined yet. For this reason the TLX LBD domain was estimated using secondary structure prediction (http://www.predictprotein.org) together with structural information from LBD crystal structures of other NRs (e.g. Androgen Receptor LBD, PDB 1T5Z [29]; COUP-TFII, PDB 3CJW [30]–[31]).

**Figure 1:**
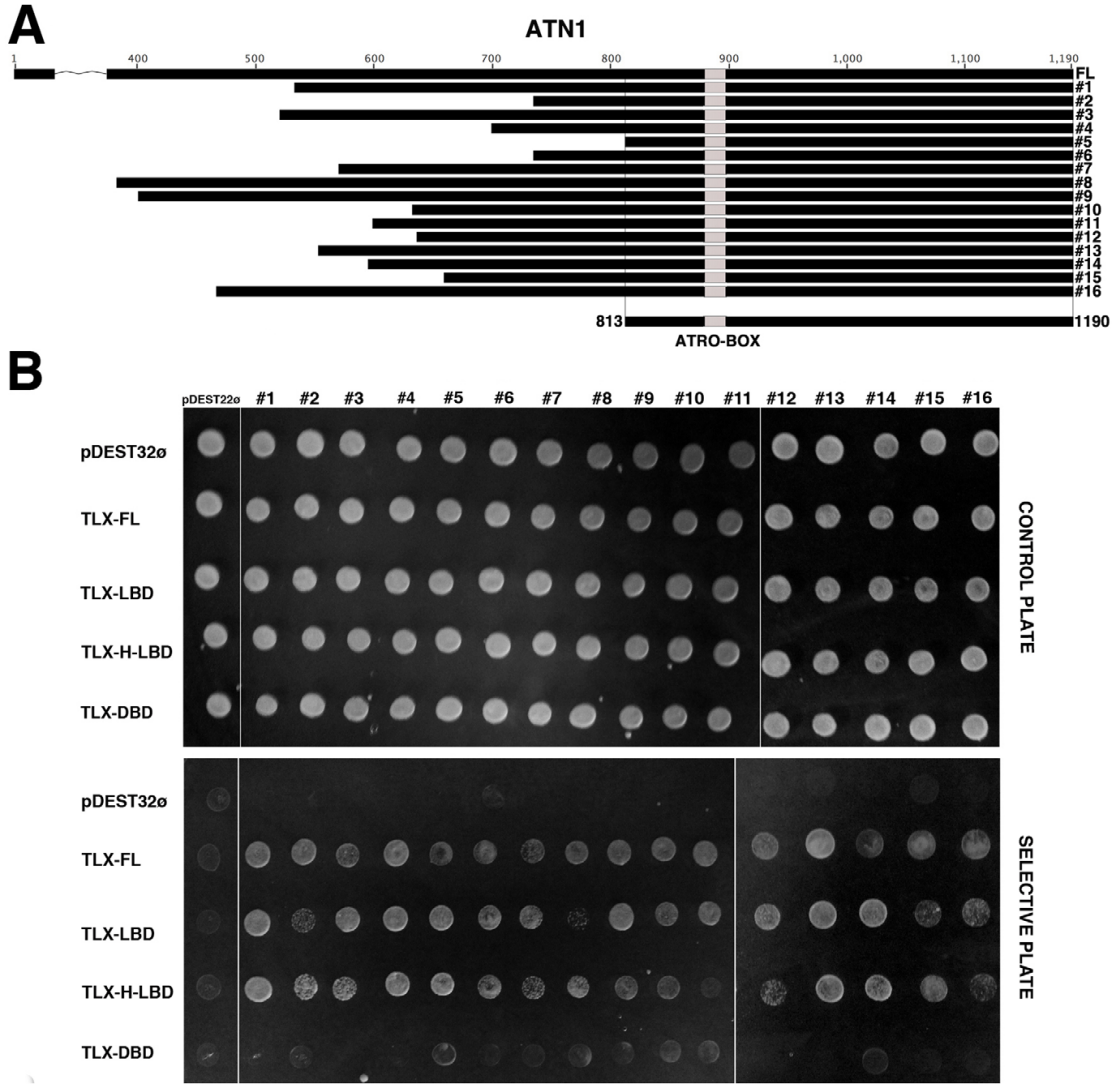
Human TLX recruits ATN1. Y2H screens using TLX-FL and TLX-LBD domain as baits against an adult brain cDNA library identified ATN1 as a TLX-interactor. (A) Schematic diagram of the identified ATN1 clones. All clones share a 378 amino acid overlapping region (residues 813–1190) featuring the ATRO-BOX region. (B) Validation of the interaction between TLX and the identified ATN1 clones by forward one-to-one Y2H assay. TLX constructs FL-TLX (1–385), TLX-LBD (172–385), TLX-H-LBD (94–385) and TLX-DBD (1–95) (baits) were tested for interaction with the ATN1 clones identified (preys). Yeast transformants were plated on a control plate (lacking Trp and Leu) and plated on a selective plate (lacking Trp, Leu, His supplemented with 50 mM 3AT).

Our screenings with the two different baits (FL-TLX and TLX-LBD) yielded a large number of clones that were identified as overlapping sequences of ATN1 (Figure 1A and Text S1-A). The region shared by all ATN1 clones comprises residues 813 to 1190, which includes the so-called ATRO-Box and is in good agreement with the previously defined TLX-binding region in ATN1 (residues 800–1000) [16] [18] [23]–[24] (Figure 1A). The identification of ATN1 as a TLX interactor confirmed the functionality of our bait constructs and Y2H screens. All identified ATN1 clones were also validated in one-to-one Y2H assays against all TLX constructs. The interaction ATN1-TLX only occurred in the TLX constructs featuring a LBD domain and was lost when TLX-NT-DBD construct was tested (Figure 1B).

**Figure 2:**
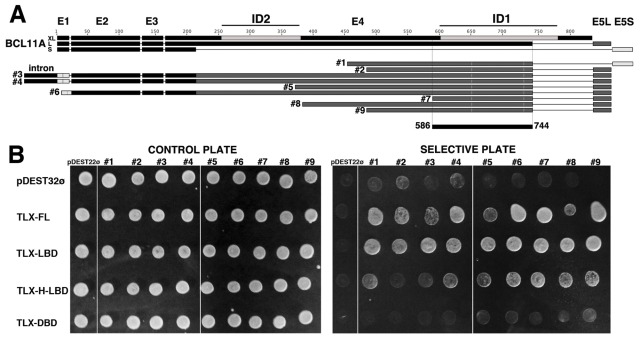
The oncoprotein BCL11A binds to human TLX. (A) Schematic diagram of the different isoforms of BCL11A (XL, L, S) and corresponding exons (1, E2, E3, E4, E5L, E5S). All BCL11A identified clones share a 159 amino acid overlapping region (residues 586–744), whose amino acid sequence is shown. Previously BCL11A regions identified to bind to COUP-FT and named ID-1 and ID-2 are highlighted in light grey. Clone 1 features a novel combination of exons, while clones 3, and 4 contain an intronic sequence. All the identified clones contain ID-2, but only some of them also have ID-2 as well. (B) Validation of the interaction between TLX and the identified BCL11A clones by forward one-to-one Y2H assay. TLX constructs FL-TLX (1–385), TLX-LBD (172–385), TLX-H-LBD (94–385) and TLX-DBD (1–95) (baits) were tested for interaction with the BCL11A clones identified (preys). Yeast transformants were plated on a control plate (lacking Trp and Leu) and plated on a selective plate (lacking Trp, Leu, His supplemented with 50 mM 3AT).

When we analyzed the remaining clones, we identified the oncoprotein BCL11A as a novel interactor of TLX (Figure 2A and Text S1-B). We obtained several overlapping clones of BCL11A in independent screens using both baits (FL-TLX and TLX-LBD). All BCL11A clones shared a region comprising residues 586 to 744 (Figure 2A and Text S1-B). Secondary structure predictions indicate that this fragment might acquire an α-helical structure flanked by two short ß-sheets. This region contained the COUP-TFII interaction domain ID1 (Figure 2A) [27]. A second BCL11A domain that was described to interact with COUP-TFII and termed ID2 (residues 264–378) [27]–[28], was only present in some of the TLX-interacting clones (Figure 2A).

BCL11A has several isoforms [32]–[35] (Figure 2A and Text S1-B). All but one of the identified BCL11A clones correspond to the BCL11A-L isoform (Figure 2A). Clone #1 (Figure 2A and Text S1-B) features an unreported combination of sequences find in both BCL11A-L and S isoforms, suggesting that it might be a novel BCL11A isoform (Personal communication with Dr. Jian Xu, Harvard, USA). We have deposited this sequence as BCL11A-M in the NCBI GenBank database with the accession number JN852960 (http://www.ncbi.nlm.nih.gov/genbank/). Additionally, clones 3 and 4 present intronic regions at their N-termini indicating that they may be unspliced forms (Figure 2A and Text S1-B). BCL11A clones were validated as TLX interactors in one-to-one Y2H assays and the interaction seems to be primarily LBD-driven (Figure 2B).

### TLX Interacts with BCL11A after Co-Expression in Human Cells

**Figure 3:**
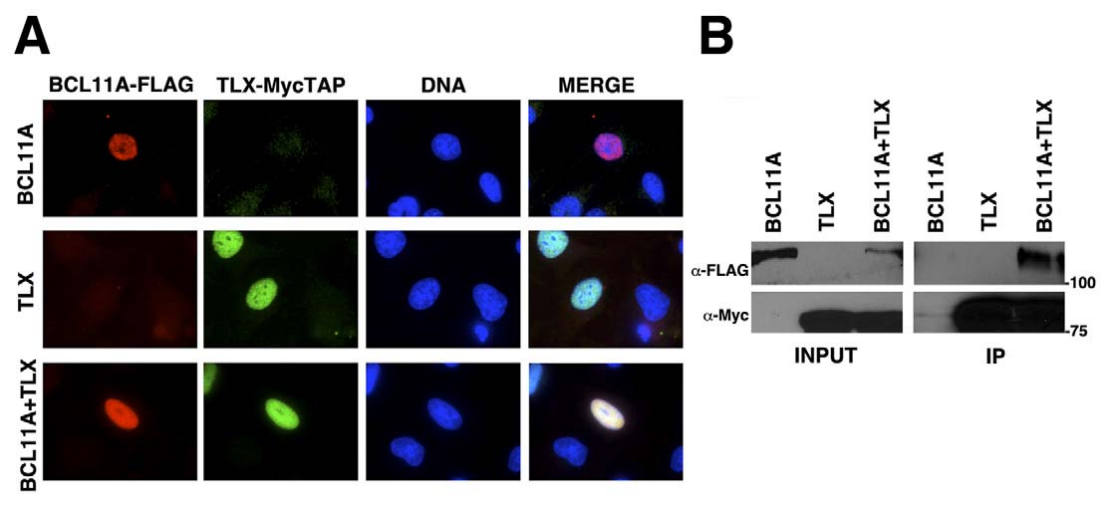
BCL11A-XL interaction with TLX-FL in human cells. (A) Transient transfection of U2OS cells with MycTAP-tagged TLX-FL and FLAG-tagged BCL11A-XL indicate that both proteins exhibit a nuclear localization as confirmed by immunofluorescence microscopy. (B) After transient transfection of HEK293 cells, pull-down of TLX-FL-MycTAP co-precipitated FLAG-BCL11A-XL, thus confirming the interaction between both proteins observed in the Y2H assay.

To confirm the interaction between TLX and BCL11A in human cells we performed transient transfections of MycTAP-tagged TLX-FL and FLAG-tagged BCL11A-XL into human U2OS and HEK293 cells (Figure 3). Analysis of transfected U2OS cells by immunofluorescence microscopy confirmed that both constructs displayed the expected nuclear localization (Figure 3A). Nuclear localization was observed for both proteins alone and in combination, indicating that both proteins localize to the nucleus independent of the presence of the binding partner. Most importantly, after transient co-expression in HEK293 cells pull-down of TLX-FL-MycTAP co-precipitated FLAG-BCL11A-XL (Figure 3B), confirming the interaction between both proteins observed in the Y2H assay.

### BCL11A Potentiates the Transrepressive Activity of TLX

**Figure 4:**
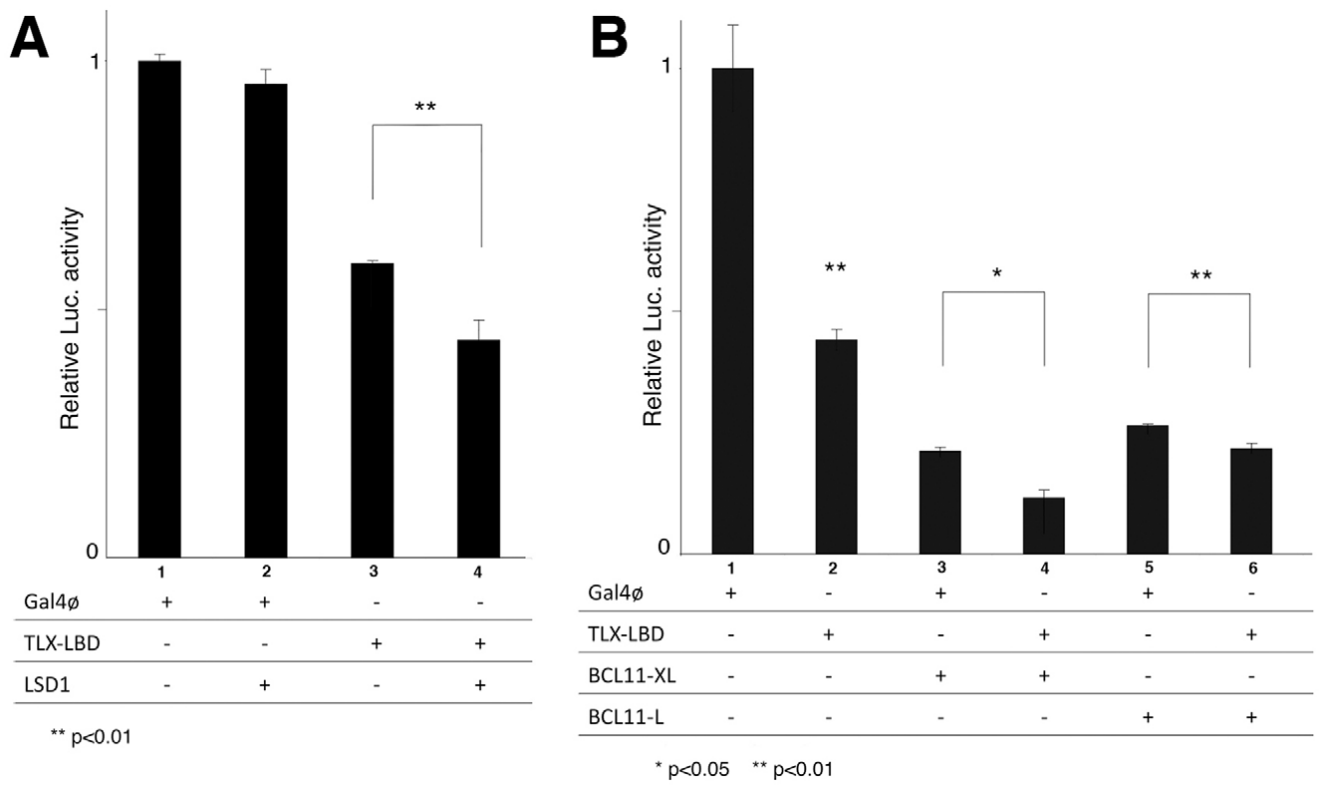
BCL11A is a TLX corepressor. (A) Chromatin luciferase [Luc] assay showing corepressor activity of LSD1 with TLX as previosly published [18]. 293F cells, which have a stably integrated pGL4.31 reporter gene, were transiently transfected with pM or pM TLX LBD vector (200 ng each) and with 400 ng of pcDNA empty vector, and pCMX-FLAG LSD1. (B) Chromatin luciferase [Luc] assay showing corepressor activity of BCL11A with TLX. 293F cells, which have a stably integrated pGL4.31 reporter gene, were transiently transfected with pM or pM TLX LBD vector (200 ng each) and with 400 ng of pcDNA empty vector, pEF1a-BCL11-XL or pEF1a-BCL11-L. The P values were calculated by Student’s t test (n = 3).

We next investigated the functional significance of the interaction between TLX and BCL11A in TLX transrepression activity using an *in vitro* luciferase reporter assay [18]. We used HEK293F cells, which have a stably integrated pGL4.31 reporter gene containing five GAL4 upstream activation sequence sites in the luciferase gene promoter, which were transiently transfected with TLX-LBD and coregulators, alone or in combination. Transfection of TLX-LBD alone repressed reporter gene activity, while co-transfection of TLX-LBD with LSD1, a known TLX coregulator, potentiated the transrepressive activity of TLX as previously described [18] (Figure 4A). When combining TLX-LBD with BCL11-XL and L isoforms a similar potentiation of TLX transrepression activity was observed (Figure 4B). Furthermore, transfection of BCL11A isoforms alone also repressed the luciferase reporter (Figure 4B). Together our data suggests that BCL11A functions as a TLX coregulator that represses transcriptional activity in TLX-dependent and TLX-independent ways.

## Discussion

TLX participation in key brain functions and the maintenance of adult NSC stemness has solid experimental documentation [3]–[7] [9] [13] but the molecular mechanism by which this orphan NR exerts its functions is still not understood. Little is known about potential TLX regulators and for this reason the identification and detailed mapping of TLX interactors is crucial for understanding brain development, adult neurogenesis and brain tumor formation.

Using Y2H screening we identified several overlapping fragments of ATN1, a known TLX interactor [16], which validated our experimental setup. In addition, we identified the oncoprotein BCL11A as a novel TLX interactor and confirmed this interaction by expression and coprecipitation in human cells. ATN1 and BCL11A do not belong to the same family of proteins but both have been described to act as transcriptional repressors [16] [21] [24] [27]–[28].

Our Y2H results corroborate previously reported data showing that TLX recruitment of ATN1 depends on its LBD as the docking domain [16] [18] [24]. Wang *et al.* identified a region in ATN1 termed the ATRO-Box, which mediates binding to TLX-LBD [24]. Strikingly, all of the clones identified as TLX interactors in our study contain the ATRO-Box.

TLX physical interaction with BCL11A has not been previously described. However, BCL11A has been reported to bind to all three COUP-TF family members, which are NRs highly homologous to TLX. The interaction between BCL11A and COUP-TFII involves the two independent interaction domains ID1 and ID2 of BCL11A [27]–[28]. In our assays the presence of a region comprising ID1 was sufficient to mediate interaction with the TLX and we found that this interaction occurred with the LBD of TLX.

Furthermore, we have shown that BCL11A potentiates TLX transrepressive function in an *in vitro* luciferase assay. BCL11A has previously been shown to repress transcription by two mechanisms: via direct binding to specific GC-rich DNA sequences and via interaction with the COUP-TF members [28]. The first mechanism of action was reported to be relevant to the physiological and/or pathological actions of BCL11A in cells of the haematopoietic and immune systems [32]–[34] [36], which do not express COUP-TF family members [27], but the latter is of relevance in the brain. BCL11A has also been shown to be functional in the brain [27]–[28] [35] [37]–[39]. Whereas the S isoform of BCL11A was widely expressed in different regions of the rat brain, the L isoform was more restrictedly expressed in the cerebral cortex, hippocampus, and olfactory bulb [38]. Almost all of our clones contain sequences matching the L isoform, while three additional sequences might represent previously undescribed BCL11A variants. Thus, BCL11A expression patterns would be consistent with a functional interaction with TLX, which also shows brain-specific expression. TLX association with BCL11A might provide a new therapeutic approach for brain tumors.

In summary, we have identified and validated the oncoprotein BCL11A as a novel TLX-interactor, and have shown that BCL11A enhances TLX-dependent transrepression. However, as previously reported, BCL11A acts also as a nuclear receptor-independent transcription factor. Our data and the previous report that COUP-TFs recruits BCL11A indicate a more widespread coregulatory role for BCL11A in the NR superfamily. Whether TLX and BCL11A might also cooperate in transcriptional regulation in the hematopoietic system needs further investigation but earlier studies suggested a role for this orphan NR in human B-cell leukemia [40].

## Materials and Methods

### Molecular Biology

#### Y2H Constructs

The Invitrogen Gateway system was used to clone TLX constructs into pDEST32 bait vector. FL-TLX (1–385), TLX-H-LBD (94–385), TLX-NT-DBD (1–95) and TLX-LBD (172–385) were first cloned into the Gateway entry pDONR221/ZEO vector and then subcloned into the pDEST32 vector via recombination. TLX constructs were amplified from a TLX full-length cDNA clone using standard PCR using the following primers (Sigma-Aldrich):

PCR1_TLX-LBD

5′- GGC TTC GAA AAC CTG TAC TTC CAG GGC ACC CCA ATG TAT CTC TAT GAA GT- 3′

PCR1_TLX-H-LBD:

5′- GGC TTC GAA AAC CTG TAC TTC CAG GGC GAG CGG GGG CCT CG-3′

PCR1 common forward primer for FL-TLX and TLX-NT-DBD:

5′- GGC TTC GAA AAC CTG TAC TTC CAG GGC ATG AGC AAG CCA GCC GGA TC -3′

PCR-1 common reverse primer for FL-TLX, TLX-H-LBD and TLX-LBD:

5′- CAA GAA AGC TGG GTT CTA GAT ATC ACT GGA TTT GTA CAT ATC TGA AAG -3′

PCR-1 reverse primer for TLX-NT-DBD

5′- CAA GAA AGC TGG GTT CTA GTG CTG CAC GGC GTC TTT GTT -3′

PCR2 Gateway Invitrogen primers:

5′- GGGG ACA AGT TTG TAC AAA AAA GCA GGC TTC GAA AAC CTG TAC TTC CAG -3′

5′- GGGG AC CAC TTT GTA CAA GAA AGC TGG GTT CTA GAT ATC ACT GGA-3′

PCR1 added at the 5′ end a TEV cleavage site (residues ENYLFQG) and the attB1 site while at the 3′ end the attB2 site was added. The attB sites were required to recombine with the entry vector pDONR221/ZEO vector during the so-called BP reaction (following manufacturer protocol). Integrity of insert sequence was verified by means of bi-directional sequencing (Macrogen, Inc).

Once we obtained our entry vectors, the different constructs of human TLX were transferred to pDEST32 vector using the so-called LR reaction creating the Y2H baits, which consist of a GAL4 DNA binding domain fused to the different TLX domains.

#### Luciferase assay constructs

The following constructs GAL4-TLX (pM TLX-LBD (172–385), empty GAL4 vector, pcDNA empty vector, and pCMX-FLAG LSD1 have been previously described [18] and pCMX-FLAG LSD1 [41]. The pEF1a-BCL11-XL, and pEF1a-BCL11-L have been also previously described ([33]).

#### Co-immunoprecipitation constructs

FL-TLX was also cloned into a vector containing the myc-TAP cassette at the *C*-terminus as previously described [42]. FL-TLX was introduced between the FseI/AscI restriction sites.

#### Immunofluorescence constructs

FL-TLX- mycTAP and pEF1a-BCL11-XL with FLAG were used.

### Antibodies

Rabbit anti-myc antibody was from Santa Cruz (SC-789) and mouse anti-FLAG M2 monoclonal antibody was from Sigma (F1804). Secondary anti-rabbit (111-035-003) and anti-mouse (115-035-003) were from Jackson ImmunoResearch.

#### Yeast transformation

Mav203 yeast strain transformations were performed using the LiAcO/sperm salmon (SS) carrier DNA/PEG method as described in ProQuest Two-Hybrid System user manual (Invitrogen). Yeast cells were made competent and then suspended with bait and prey vectors and an excess of carrier DNA in a LiAcO solution with PEG, and incubated at 30°C. After incubation, DMSO was added and the cells were heat shocked at 42°C (50 min in the case of library scale transformation or 7 minutes in the case of small scale transformation). Transformed yeast was then plated on the appropriate SD medium to select transformants (SD-L-T; lacking Trp and Leu and SD-L-T-H; lacking Trp, Leu and His).

#### Y2H screens with adult brain cDNA library

Y2H library screens were performed using an adult human brain cDNA prey library (ProQuest, Invitrogen 113746-027) against FL-TLX (1–385) and TLX-LBD (172–385). Bait TLX-LBD was cloned, as described previously, into the Y2H destination vector pDEST32 by *Gateway* recombinational cloning (ProQuest System, Invitrogen). Bait plasmid was transformed into a Mav203 yeast strain in a first small scale transformation step and plated into plates lacking Leu (SD-L) to select bait transformed yeast. After 3 days incubation at 30°C, a replica clean of the transformants was made and plates were incubated for 3 additional days at 30°C. Yeast containing bait were then transformed against a human brain cDNA prey library (human brain cDNAs fused to Gal4 Activation Domain (AD), Invitrogen) and plated into selective plates: SD1 (SD lacking Leu, Trp and His+20 mM 3-amino-1,2,4-triazole (3AT)) and SD2 (SD lacking Leu, Trp and His+50 mM 3AT). After 5 days incubation at 30°C, positive growing colonies were picked up and cultured in prey selective liquid medium (SD-T, lacking Trp). Prey plasmids DNAs were then extracted from cultures and shuttled in *E. coli* DH5a strain to enable DNA sequencing (Macrogen, Inc) using the following primers (Sigma-Aldrich): 5′ TATAACGCGTTTGGAATCACT 3 and 5′′ TAAATTTCTGGCAAGGTAGAC ′3. Finally, gene identification by BLASTp (NCBI) was performed.

#### Y2H validations using forward one-to-one Y2H assays

Bait and prey plasmids were pair-wise co-transformed into Mav203 yeast strain in a 96-well array format. Co-transformed cells were plated onto selective SD-L-T plates and incubated for 48 hours at 30°C. After a colony replica clean plating, co-transformant arrays were replicated onto different selective plates (SD1, SD2) to detect HIS3 reporters induction.

#### Luciferase assays

We used the stable cell line HEK 293F-pGL4.31, which has been previously described [18]. This cell line expresses consitutively pGL4.31 reporter plasmid, which contains five GAL4 response elements upstream of a minimal promoter.

HEK 293F-pGL4.31 cell line was maintained in Dulbecco’s modified Eagle, 4.5 g/liter D-glucose Medium (Gibco) containing 10% fetal bovine serum, 0.58 g/liter L-glutamine, 0.11 g/liter sodium pyruvate, 100 u/ml penicillin, 100 mg/ml streptomycin and 200 ng/uL Hygromicin. Twenty-four hours before transfection, cells were collected in fresh medium seeded in 12-well culture plates (Corning) at a density of 3×10^5^ cells per well. They were transfected using FuGENE HD reagent (Promega) as described by the manufacturer. The DNA mixture was composed of 0,1 ng/well of Renilla-LUC; 200 ng/well of GAL4-TLX-LBD (172–385) or empty control vector; and 400 ng/well of pEF1a-BCL11 isoforms XL, L or S; or empty control vector. After twenty-four hours, cells were washed twice with phosphate-buffered saline and lysed in 200 uL of passive lysis buffer (Promega). LUC and Renilla-LUC activities were measured on 100 uL of the extracts in a GloMax 96 Microplate Luminometer (Promega) using the Dual-Luciferase Reporter Assay System (Promega), according to the manufacturer’s instructions.

### Immunoprecipitation

For immunoprecipitation of FLAG-tagged BCL11A-XL and MycTAP-tagged TLX-FL, transfected human embryonic kidney cells HEK293 cells were washed in PBS and lysed (50 mM HEPES, pH 7.5, 150 mM NaCl, 1 mM MgCl2, 1 mM EGTA, 0.5% NP-40, protease inhibitors) for 10 min on ice. After centrifugation for 15 min at 16,000 *g* at 4°C cleared lysates were incubated with anti-GFP antibodies for 1 h at 4°C. Sepharose Protein G beads (GE Healthcare) were added and the mixture was incubated for an additional hour at 4°C. The beads were pelleted and washed three times with lysis buffer. Samples were prepared for SDS-PAGE by boiling in sample buffer.

### Western Blotting

Cells were washed in PBS and lysed (50 mM HEPES pH 7.5, 150 mM NaCl, 1 mM MgCl2, 1 mM EGTA, 0.5% NP-40, protease inhibitors) on ice. Cleared extracts were prepared by centrifugation and subjected to SDS-PAGE. Proteins were transferred to nitrocellulose membranes and probed with antibodies.

### Fluorescence Microscopy

Human osteosarcoma U2OS cells grown on coverslips were fixed in methanol at −20°C for at least 5 min and processed for immunofluorescence. Alternatively, cells were fixed in PBS containing 4% paraformaldehyde, 0.05% glutaraldehyde, and 0.1% Triton X-100 for 15 min at RT.

Fixed cells were blocked in PBS-BT (1x PBS, 0.1% Triton X-100, and 3% BSA) and incubated with antibodies in the same buffer. Images were acquired with an Orca AG camera (Hamamatsu, Bridgewater, NJ) on a Leica DMI6000B microscope equipped with 1.4 NA 63x and 100x oil immersion objectives. AF6000 software (Leica, Wetzlar, Germany) was used for image acquisition. For further image processing and quantification of fluorescence intensities ImageJ software was used.

## Supporting Information

Text S1 (A) ATN1 Clones Obtained. From several independent Y2H screens 7 clones of ATN1 have been identified using TLX-LBD (172–385) as a bait, and a total of 9 ATN using TLX-FL (1–385) as bait. All 16 clones contain the ATRO-Box region, which has been previously described to interacts with TLX. The residue sequence of each clone is shown. The common region between all ATN1 clones is underlined, while the so-called Atro-box is highlighted green. (B) BCL11A Clones Obtained. From several independent Y2H screens 4 clones of BCL11A have been identified using TLX-LBD (172–385) as a bait, and a total of 5 BCL11A using TLX-FL (1–385) as bait. The detailed sequence and description of these clones are indicated. The reported isoforms are produced by alternative splicing of 7 different exons (E1, E2, E-XS, E3, E4, E5-S and E5-L). The residue sequence belonging to E2, E3, and E4 is highlighted in blue, while the one belonging to E5-S is highlighted in orange and the one belonging to E5-L is highlighted in green. A novel isoform of BCL11A that we called isoform M, has been identified and corresponds to clone #1. The rest of the clones correspond only to isoform L.
(DOCX)
