# Supplementary material for: The Oncoprotein BCL11A Binds to Orphan Nuclear Receptor TLX and Potentiates its Transrepressive Function
Source: PLoS One. 2012 Jun 4;7(6):e37963. doi: 10.1371/journal.pone.0037963 (PMC3366998; doi:10.1371/journal.pone.0037963)
Supplement: Text S1 — (A) ATN1 Clones Obtained. From several independent Y2H screens 7 clones of ATN1 have been identified using TLX-LBD (172–385) as a bait, and a total of 9 ATN using TLX-FL (1–385) as bait. All 16 clones contain the ATRO-Box region, which has been previously described to interacts with TLX. The residue sequence of each clone is shown. The common region between all ATN1 clones is underlined, while the so-called Atro-box is highlighted green. (B) BCL11A Clones Obtained. From several independent Y2H screens 4 clones of BCL11A have been identified using TLX-LBD (172–385) as a bait, and a total of 5 BCL11A using TLX-FL (1–385) as bait. The detailed sequence and description of these clones are indicated. The reported isoforms are produced by alternative splicing of 7 different exons (E1, E2, E-XS, E3, E4, E5-S and E5-L). The residue sequence belonging to E2, E3, and E4 is highlighted in blue, while the one belonging to E5-S is highlighted in orange and the one belonging to E5-L is highlighted in green. A novel isoform of BCL11A that we called isoform M, has been identified and corresponds to clone #1. The rest of the clones correspond only to isoform L. (DOCX) [file pone.0037963.s001.docx]

**SUPPORTING INFORMATION TEXT S1**

**(A) ATN1 Clones Obtained.** From several independent Y2H screens 7 clones of ATN1 have been identified using TLX-LBD (172-385) as a bait, and a total of 9 ATN using TLX-FL (1-385) as bait. All 16 clones contain the ATRO-Box region, which has been previously described to interacts with TLX. The residue sequence of each clone is shown. The common region between all ATN1 clones is underlined, while the so-called Atro-box is highlighted green.

**ATN1#1:** Residues 533-1190.

_571_ S P S L G S L R P Y P P G P A H L P P P H S Q V S Y S Q A G P N G P P V S S S S N S S S S T S Q G S Y P C S H P S P S Q G P Q G A P Y P F P P V P T V T T S S A T L S T V I A T V A S S P A G Y K T A S P P G P P P Y G K R A P S P G A Y K T A T P P G Y K P G S P P S F R T G T P P G Y R G T S P P A G P G T F K P G S P T V G P G P L P P A G P S G L P S L P P P P A A P A S G P P L S A T Q I K Q E P A E E Y E T P E S P V P P A R S P S P P P K V V D V P S H A S Q S A R F N K H L D R G F N S C A R S D L Y F V P L E G S K L A K K R A D L V E K V R R E A E Q R A R E E K E R E R E R E R E K E R E R E K E R E L E R S V K L A Q E G R A P V E C P S L G P V P H R P P F E P G S A V A T V P P Y L G P **D T P A L R T L S E Y A R P H V** M S P G N R N H P F Y V P L G A V D P G L L G Y N V P A L Y S S D P A A R E R E R E A R E R D L R D R L K P G F E V K P S E L E P L H G V P G P G L D P F P R H G G L A L Q P G P P G L H P F P F H P S L G P LE R E R L A L A A G P A L R PD M S YA E R L A A E R Q H A E R V A A L G N D P L A R L Q M L N V T P H H H Q H S H I H S H L H L H Q Q D A I H A A S A S V H P L I D P L A S G S H L T R I P Y P A G T L P N P L L P H P L H E N E V L R H Q L F A A P Y RD L P A S L S A P M S A A H Q L Q A M H A Q S A E L Q R L A L E Q Q Q W L H A H H P L H S V P L P A Q E D Y Y S H L K K E S D K P L _1190_

**ATN1#2:** Residues 735-1190.

_735_ Y E T P E S P V P P A R S P S P P P K V V D V P S H A S Q S A R F N K H L D R G F N S C A R S D L Y F V P L E G S K L A K K R A D L V E K V R R E A E Q R A R E E K E R E R E R E R E K E R E R E K E R E L E R S V K L A Q E G R A P V E C P S L G P V P H R P P F E P G S A V A T V P P Y L G P **D T P A L R T L S E Y A R P H V** M S P G N R N H P F Y V P L G A V D P G L L G Y N V P A L Y S S D P A A R E R E R E A R E R D L R D R L K P G F E V K P S E L E P L H G V P G P G L D P F P R H G G L A L Q P G P P G L H P F P F H P S L G P LE R E R L A L A A G P A L R PD M S YA E R L A A E R Q H A E R V A A L G N D P L A R L Q M L N V T P H H H Q H S H I H S H L H L H Q Q D A I H A A S A S V H P L I D P L A S G S H L T R I P Y P A G T L P N P L L P H P L H E N E V L R H Q L F A A P Y RD L P A S L S A P M S A A H Q L Q A M H A Q S A E L Q R L A L E Q Q Q W L H A H H P L H S V P L P A Q E D Y Y S H L K K E S D K P L _1190_

**ATN1#3:** Residues 521-1190.

_521_ G G S S H H A H P Y A M S P S L G S L R P Y P P G P A H L P P P H S Q V S Y S Q A G P N G P P V S S S S N S S S S T S Q G S Y P C S H P S P S Q G P Q G A P Y P F P P V P T V T T S S A T L S T V I A T V A S S P A G Y K T A S P P G P P P Y G K R A P S P G A Y K T A T P P G Y K P G S P P S F R T G T P P G Y R G T S P P A G P G T F K P G S P T V G P G P L P P A G P S G L P S L P P P P A A P A S G P P L S A T Q I K Q E P A E E Y E T P E S P V P P A R S P S P P P K V V D V P S H A S Q S A R F N K H L D R G F N S C A R S D L Y F V P L E G S K L A K K R A D L V E K V R R E A E Q R A R E E K E R E R E R E R E K E R E R E K E R E L E R S V K L A Q E G R A P V E C P S L G P V P H R P P F E P G S A V A T V P P Y L G P **D T P A L R T L S E Y A R P H V** M S P G N R N H P F Y V P L G A V D P G L L G Y N V P A L Y S S D P A A R E R E R E A R E R D L R D R L K P G F E V K P S E L E P L H G V P G P G L D P F P R H G G L A L Q P G P P G L H P F P F H P S L G P LE R E R L A L A A G P A L R PD M S YA E R L A A E R Q H A E R V A A L G N D P L A R L Q M L N V T P H H H Q H S H I H S H L H L H Q Q D A I H A A S A S V H P L I D P L A S G S H L T R I P Y P A G T L P N P L L P H P L H E N E V L R H Q L F A A P Y RD L P A S L S A P M S A A H Q L Q A M H A Q S A E L Q R L A L E Q Q Q W L H A H H P L H S V P L P A Q E D Y Y S H L K K E S D K P L _1190_

**ATN1#4:** Residues 700-1190.

_700_ A G P S G L P S L P P P P A A P A S G P P L S A T Q I K Q E P A E E Y E T P E S P V P P A R S P S P P P K V V D V P S H A S Q S A R F N K H L D R G F N S C A R S D L Y F V P L E G S K L A K K R A D L V E K V R R E A E Q R A R E E K E R E R E R E R E K E R E R E K E R E L E R S V K L A Q E G R A P V E C P S L G P V P H R P P F E P G S A V A T V P P Y L G P **D T P A L R T L S E Y A R P H V** M S P G N R N H P F Y V P L G A V D P G L L G Y N V P A L Y S S D P A A R E R E R E A R E R D L R D R L K P G F E V K P S E L E P L H G V P G P G L D P F P R H G G L A L Q P G P P G L H P F P F H P S L G P LE R E R L A L A A G P A L R PD M S YA E R L A A E R Q H A E R V A A L G N D P L A R L Q M L N V T P H H H Q H S H I H S H L H L H Q Q D A I H A A S A S V H P L I D P L A S G S H L T R I P Y P A G T L P N P L L P H P L H E N E V L R H Q L F A A P Y RD L P A S L S A P M S A A H Q L Q A M H A Q S A E L Q R L A L E Q Q Q W L H A H H P L H S V P L P A Q E D Y Y S H L K K E S D K P L _1190_

**ATN1#5:** Residues 813-1190

_813_ E E K E R E R E R E R E K E R E R E K E R E L E R S V K L A Q E G R A P V E C P S L G P V P H R P P F E P G S A V A T V P P Y L G P **D T P A L R T L S E Y A R P H V** M S P G N R N H P F Y V P L G A V D P G L L G Y N V P A L Y S S D P A A R E R E R E A R E R D L R D R L K P G F E V K P S E L E P L H G V P G P G L D P F P R H G G L A L Q P G P P G L H P F P F H P S L G P LE R E R L A L A A G P A L R PD M S YA E R L A A E R Q H A E R V A A L G N D P L A R L Q M L N V T P H H H Q H S H I H S H L H L H Q Q D A I H A A S A S V H P L I D P L A S G S H L T R I P Y P A G T L P N P L L P H P L H E N E V L R H Q L F A A P Y RD L P A S L S A P M S A A H Q L Q A M H A Q S A E L Q R L A L E Q Q Q W L H A H H P L H S V P L P A Q E D Y Y S H L K K E S D K P L _1190_

**ATN1#6:** Residues 735-1190.

_735_ Y E T P E S P V P P A R S P S P P P K V V D V P S H A S Q S A R F N K H L D R G F N S C A R S D L Y F V P L E G S K L A K K R A D L V E K V R R E A E Q R A R E E K E R E R E R E R E K E R E R E K E R E L E R S V K L A Q E G R A P V E C P S L G P V P H R P P F E P G S A V A T V P P Y L G P **D T P A L R T L S E Y A R P H V** M S P G N R N H P F Y V P L G A V D P G L L G Y N V P A L Y S S D P A A R E R E R E A R E R D L R D R L K P G F E V K P S E L E P L H G V P G P G L D P F P R H G G L A L Q P G P P G L H P F P F H P S L G P LE R E R L A L A A G P A L R PD M S YA E R L A A E R Q H A E R V A A L G N D P L A R L Q M L N V T P H H H Q H S H I H S H L H L H Q Q D A I H A A S A S V H P L I D P L A S G S H L T R I P Y P A G T L P N P L L P H P L H E N E V L R H Q L F A A P Y RD L P A S L S A P M S A A H Q L Q A M H A Q S A E L Q R L A L E Q Q Q W L H A H H P L H S V P L P A Q E D Y Y S H L K K E S D K P L _1190_

**ATN1#7:** Residues 571-1190.

_571_ S S N S S S S T S Q G S Y P C S H P S P S Q G P Q G A P Y P F P P V P T V T T S S A T L S T V I A T V A S S P A G Y K T A S P P G P P P Y G K R A P S P G A Y K T A T P P G Y K P G S P P S F R T G T P P G Y R G T S P P A G P G T F K P G S P T V G P G P L P P A G P S G L P S L P P P P A A P A S G P P L S A T Q I K Q E P A E E Y E T P E S P V P P A R S P S P P P K V V D V P S H A S Q S A R F N K H L D R G F N S C A R S D L Y F V P L E G S K L A K K R A D L V E K V R R E A E Q R A R E E K E R E R E R E R E K E R E R E K E R E L E R S V K L A Q E G R A P V E C P S L G P V P H R P P F E P G S A V A T V P P Y L G P **D T P A L R T L S E Y A R P H V** M S P G N R N H P F Y V P L G A V D P G L L G Y N V P A L Y S S D P A A R E R E R E A R E R D L R D R L K P G F E V K P S E L E P L H G V P G P G L D P F P R H G G L A L Q P G P P G L H P F P F H P S L G P LE R E R L A L A A G P A L R PD M S YA E R L A A E R Q H A E R V A A L G N D P L A R L Q M L N V T P H H H Q H S H I H S H L H L H Q Q D A I H A A S A S V H P L I D P L A S G S H L T R I P Y P A G T L P N P L L P H P L H E N E V L R H Q L F A A P Y RD L P A S L S A P M S A A H Q L Q A M H A Q S A E L Q R L A L E Q Q Q W L H A H H P L H S V P L P A Q E D Y Y S H L K K E S D K P L _1190_

**ATN1#8:** Residues 383-1190.

_383_ A A S S S S S S S S S S A S P F P A S Q A L P S Y P H S F P P P T S L S V S N Q P P K Y T Q P S L P S Q A V W S Q G P P P P P P Y G R L L A N S N A H P G P F P P S T G A Q S T A H P P V S T H H H H H Q Q Q Q Q Q Q Q Q Q Q Q Q Q Q Q Q Q Q Q Q Q Q H H G N S G P P P P G A F P H P L E G G S S H H A H P Y A M S P S L G S L R P Y P P G P A H L P P P H S Q V S Y S Q A G P N G P P V S S S S N S S S S T S Q G S Y P C S H P S P S Q G P Q G A P Y P F P P V P T V T T S S A T L S T V I A T V A S S P A G Y K T A S P P G P P P Y G K R A P S P G A Y K T A T P P G Y K P G S P P S F R T G T P P G Y R G T S P P A G P G T F K P G S P T V G P G P L P P A G P S G L P S L P P P P A A P A S G P P L S A T Q I K Q E P A E E Y E T P E S P V P P A R S P S P P P K V V D V P S H A S Q S A R F N K H L D R G F N S C A R S D L Y F V P L E G S K L A K K R A D L V E K V R R E A E Q R A R E E K E R E R E R E R E K E R E R E K E R E L E R S V K L A Q E G R A P V E C P S L G P V P H R P P F E P G S A V A T V P P Y L G P **D T P A L R T L S E Y A R P H V** M S P G N R N H P F Y V P L G A V D P G L L G Y N V P A L Y S S D P A A R E R E R E A R E R D L R D R L K P G F E V K P S E L E P L H G V P G P G L D P F P R H G G L A L Q P G P P G L H P F P F H P S L G P LE R E R L A L A A G P A L R PD M S YA E R L A A E R Q H A E R V A A L G N D P L A R L Q M L N V T P H H H Q H S H I H S H L H L H Q Q D A I H A A S A S V H P L I D P L A S G S H L T R I P Y P A G T L P N P L L P H P L H E N E V L R H Q L F A A P Y RD L P A S L S A P M S A A H Q L Q A M H A Q S A E L Q R L A L E Q Q Q W L H A H H P L H S V P L P A Q E D Y Y S H L K K E S D K P L _1190_

**ATN1#9:** Residues 401-1190.

_401_ A S Q A L P S Y P H S F P P P T S L S V S N Q P P K Y T Q P S L P S Q A V W S Q G P P P P P P Y G R L L A N S N A H P G P F P P S T G A Q S T A H P P V S T H H H H H Q Q Q Q Q Q Q Q Q Q Q Q Q Q Q Q Q Q Q Q Q Q Q H H G N S G P P P P G A F P H P L E G G S S H H A H P Y A M S P S L G S L R P Y P P G P A H L P P P H S Q V S Y S Q A G P N G P P V S S S S N S S S S T S Q G S Y P C S H P S P S Q G P Q G A P Y P F P P V P T V T T S S A T L S T V I A T V A S S P A G Y K T A S P P G P P P Y G K R A P S P G A Y K T A T P P G Y K P G S P P S F R T G T P P G Y R G T S P P A G P G T F K P G S P T V G P G P L P P A G P S G L P S L P P P P A A P A S G P P L S A T Q I K Q E P A E E Y E T P E S P V P P A R S P S P P P K V V D V P S H A S Q S A R F N K H L D R G F N S C A R S D L Y F V P L E G S K L A K K R A D L V E K V R R E A E Q R A R E E K E R E R E R E R E K E R E R E K E R E L E R S V K L A Q E G R A P V E C P S L G P V P H R P P F E P G S A V A T V P P Y L G P **D T P A L R T L S E Y A R P H V** M S P G N R N H P F Y V P L G A V D P G L L G Y N V P A L Y S S D P A A R E R E R E A R E R D L R D R L K P G F E V K P S E L E P L H G V P G P G L D P F P R H G G L A L Q P G P P G L H P F P F H P S L G P LE R E R L A L A A G P A L R PD M S YA E R L A A E R Q H A E R V A A L G N D P L A R L Q M L N V T P H H H Q H S H I H S H L H L H Q Q D A I H A A S A S V H P L I D P L A S G S H L T R I P Y P A G T L P N P L L P H P L H E N E V L R H Q L F A A P Y RD L P A S L S A P M S A A H Q L Q A M H A Q S A E L Q R L A L E Q Q Q W L H A H H P L H S V P L P A Q E D Y Y S H L K K E S D K P L _1190_

**ATN1#10:** Residues 633-1190.

_633_ P P G P P P Y G K R A P S P G A Y K T A T P P G Y K P G S P P S F R T G T P P G Y R G T S P P A G P G T F K P G S P T V G P G P L P P A G P S G L P S L P P P P A A P A S G P P L S A T Q I K Q E P A E E Y E T P E S P V P P A R S P S P P P K V V D V P S H A S Q S A R F N K H L D R G F N S C A R S D L Y F V P L E G S K L A K K R A D L V E K V R R E A E Q R A R E E K E R E R E R E R E K E R E R E K E R E L E R S V K L A Q E G R A P V E C P S L G P V P H R P P F E P G S A V A T V P P Y L G P **D T P A L R T L S E Y A R P H V** M S P G N R N H P F Y V P L G A V D P G L L G Y N V P A L Y S S D P A A R E R E R E A R E R D L R D R L K P G F E V K P S E L E P L H G V P G P G L D P F P R H G G L A L Q P G P P G L H P F P F H P S L G P LE R E R L A L A A G P A L R PD M S YA E R L A A E R Q H A E R V A A L G N D P L A R L Q M L N V T P H H H Q H S H I H S H L H L H Q Q D A I H A A S A S V H P L I D P L A S G S H L T R I P Y P A G T L P N P L L P H P L H E N E V L R H Q L F A A P Y RD L P A S L S A P M S A A H Q L Q A M H A Q S A E L Q R L A L E Q Q Q W L H A H H P L H S V P L P A Q E D Y Y S H L K K E S D K P L _1190_

**ATN1#11:** Residues 599-1190.

_599_ Y P F P P V P T V T T S S A T L S T V I A T V A S S P A G Y K T A S P P G P P P Y G K R A P S P G A Y K T A T P P G Y K P G S P P S F R T G T P P G Y R G T S P P A G P G T F K P G S P T V G P G P L P P A G P S G L P S L P P P P A A P A S G P P L S A T Q I K Q E P A E E Y E T P E S P V P P A R S P S P P P K V V D V P S H A S Q S A R F N K H L D R G F N S C A R S D L Y F V P L E G S K L A K K R A D L V E K V R R E A E Q R A R E E K E R E R E R E R E K E R E R E K E R E L E R S V K L A Q E G R A P V E C P S L G P V P H R P P F E P G S A V A T V P P Y L G P **D T P A L R T L S E Y A R P H V** M S P G N R N H P F Y V P L G A V D P G L L G Y N V P A L Y S S D P A A R E R E R E A R E R D L R D R L K P G F E V K P S E L E P L H G V P G P G L D P F P R H G G L A L Q P G P P G L H P F P F H P S L G P LE R E R L A L A A G P A L R PD M S YA E R L A A E R Q H A E R V A A L G N D P L A R L Q M L N V T P H H H Q H S H I H S H L H L H Q Q D A I H A A S A S V H P L I D P L A S G S H L T R I P Y P A G T L P N P L L P H P L H E N E V L R H Q L F A A P Y RD L P A S L S A P M S A A H Q L Q A M H A Q S A E L Q R L A L E Q Q Q W L H A H H P L H S V P L P A Q E D Y Y S H L K K E S D K P L _1190_

**ATN1#12:** Residues 637-1190.

_637_ P P Y G K R A P S P G A Y K T A T P P G Y K P G S P P S F R T G T P P G Y R G T S P P A G P G T F K P G S P T V G P G P L P P A G P S G L P S L P P P P A A P A S G P P L S A T Q I K Q E P A E E Y E T P E S P V P P A R S P S P P P K V V D V P S H A S Q S A R F N K H L D R G F N S C A R S D L Y F V P L E G S K L A K K R A D L V E K V R R E A E Q R A R E E K E R E R E R E R E K E R E R E K E R E L E R S V K L A Q E G R A P V E C P S L G P V P H R P P F E P G S A V A T V P P Y L G P **D T P A L R T L S E Y A R P H V** M S P G N R N H P F Y V P L G A V D P G L L G Y N V P A L Y S S D P A A R E R E R E A R E R D L R D R L K P G F E V K P S E L E P L H G V P G P G L D P F P R H G G L A L Q P G P P G L H P F P F H P S L G P LE R E R L A L A A G P A L R PD M S YA E R L A A E R Q H A E R V A A L G N D P L A R L Q M L N V T P H H H Q H S H I H S H L H L H Q Q D A I H A A S A S V H P L I D P L A S G S H L T R I P Y P A G T L P N P L L P H P L H E N E V L R H Q L F A A P Y RD L P A S L S A P M S A A H Q L Q A M H A Q S A E L Q R L A L E Q Q Q W L H A H H P L H S V P L P A Q E D Y Y S H L K K E S D K P L _1190_

**ATN1#13:** Residues 553-1190.

_553_ H S Q V S Y S Q A G P N G P P V S S S S N S S S S T S Q G S Y P C S H P S P S Q G P Q G A P Y P F P P V P T V T T S S A T L S T V I A T V A S S P A G Y K T A S P P G P P P Y G K R A P S P G A Y K T A T P P G Y K P G S P P S F R T G T P P G Y R G T S P P A G P G T F K P G S P T V G P G P L P P A G P S G L P S L P P P P A A P A S G P P L S A T Q I K Q E P A E E Y E T P E S P V P P A R S P S P P P K V V D V P S H A S Q S A R F N K H L D R G F N S C A R S D L Y F V P L E G S K L A K K R A D L V E K V R R E A E Q R A R E E K E R E R E R E R E K E R E R E K E R E L E R S V K L A Q E G R A P V E C P S L G P V P H R P P F E P G S A V A T V P P Y L G P **D T P A L R T L S E Y A R P H V** M S P G N R N H P F Y V P L G A V D P G L L G Y N V P A L Y S S D P A A R E R E R E A R E R D L R D R L K P G F E V K P S E L E P L H G V P G P G L D P F P R H G G L A L Q P G P P G L H P F P F H P S L G P LE R E R L A L A A G P A L R PD M S YA E R L A A E R Q H A E R V A A L G N D P L A R L Q M L N V T P H H H Q H S H I H S H L H L H Q Q D A I H A A S A S V H P L I D P L A S G S H L T R I P Y P A G T L P N P L L P H P L H E N E V L R H Q L F A A P Y RD L P A S L S A P M S A A H Q L Q A M H A Q S A E L Q R L A L E Q Q Q W L H A H H P L H S V P L P A Q E D Y Y S H L K K E S D K P L _1190_

**ATN1#14:** Residues 596-1190.

_596_ G A P Y P F P P V P T V T T S S A T L S T V I A T V A S S P A G Y K T A S P P G P P P Y G K R A P S P G A Y K T A T P P G Y K P G S P P S F R T G T P P G Y R G T S P P A G P G T F K P G S P T V G P G P L P P A G P S G L P S L P P P P A A P A S G P P L S A T Q I K Q E P A E E Y E T P E S P V P P A R S P S P P P K V V D V P S H A S Q S A R F N K H L D R G F N S C A R S D L Y F V P L E G S K L A K K R A D L V E K V R R E A E Q R A R E E K E R E R E R E R E K E R E R E K E R E L E R S V K L A Q E G R A P V E C P S L G P V P H R P P F E P G S A V A T V P P Y L G P **D T P A L R T L S E Y A R P H V** M S P G N R N H P F Y V P L G A V D P G L L G Y N V P A L Y S S D P A A R E R E R E A R E R D L R D R L K P G F E V K P S E L E P L H G V P G P G L D P F P R H G G L A L Q P G P P G L H P F P F H P S L G P LE R E R L A L A A G P A L R PD M S YA E R L A A E R Q H A E R V A A L G N D P L A R L Q M L N V T P H H H Q H S H I H S H L H L H Q Q D A I H A A S A S V H P L I D P L A S G S H L T R I P Y P A G T L P N P L L P H P L H E N E V L R H Q L F A A P Y RD L P A S L S A P M S A A H Q L Q A M H A Q S A E L Q R L A L E Q Q Q W L H A H H P L H S V P L P A Q E D Y Y S H L K K E S D K P L _1190_

**ATN1#15:** Residues 660 -1190.

_660_ G S P P S F R T G T P P G Y R G T S P P A G P G T F K P G S P T V G P G P L P P A G P S G L P S L P P P P A A P A S G P P L S A T Q I K Q E P A E E Y E T P E S P V P P A R S P S P P P K V V D V P S H A S Q S A R F N K H L D R G F N S C A R S D L Y F V P L E G S K L A K K R A D L V E K V R R E A E Q R A R E E K E R E R E R E R E K E R E R E K E R E L E R S V K L A Q E G R A P V E C P S L G P V P H R P P F E P G S A V A T V P P Y L G P **D T P A L R T L S E Y A R P H V** M S P G N R N H P F Y V P L G A V D P G L L G Y N V P A L Y S S D P A A R E R E R E A R E R D L R D R L K P G F E V K P S E L E P L H G V P G P G L D P F P R H G G L A L Q P G P P G L H P F P F H P S L G P LE R E R L A L A A G P A L R PD M S YA E R L A A E R Q H A E R V A A L G N D P L A R L Q M L N V T P H H H Q H S H I H S H L H L H Q Q D A I H A A S A S V H P L I D P L A S G S H L T R I P Y P A G T L P N P L L P H P L H E N E V L R H Q L F A A P Y RD L P A S L S A P M S A A H Q L Q A M H A Q S A E L Q R L A L E Q Q Q W L H A H H P L H S V P L P A Q E D Y Y S H L K K E S D K P L _1190_

**ATN1#16:** Residues 467-1190.

_467_ G A Q S T A H P P V S T H H H H H Q Q Q Q Q Q Q Q Q Q Q Q Q Q Q Q Q Q Q Q Q Q Q H H G N S G P P P P G A F P H P L E G G S S H H A H P Y A M S P S L G S L R P Y P P G P A H L P P P H S Q V S Y S Q A G P N G P P V S S S S N S S S S T S Q G S Y P C S H P S P S Q G P Q G A P Y P F P P V P T V T T S S A T L S T V I A T V A S S P A G Y K T A S P P G P P P Y G K R A P S P G A Y K T A T P P G Y K P G S P P S F R T G T P P G Y R G T S P P A G P G T F K P G S P T V G P G P L P P A G P S G L P S L P P P P A A P A S G P P L S A T Q I K Q E P A E E Y E T P E S P V P P A R S P S P P P K V V D V P S H A S Q S A R F N K H L D R G F N S C A R S D L Y F V P L E G S K L A K K R A D L V E K V R R E A E Q R A R E E K E R E R E R E R E K E R E R E K E R E L E R S V K L A Q E G R A P V E C P S L G P V P H R P P F E P G S A V A T V P P Y L G P **D T P A L R T L S E Y A R P H V** M S P G N R N H P F Y V P L G A V D P G L L G Y N V P A L Y S S D P A A R E R E R E A R E R D L R D R L K P G F E V K P S E L E P L H G V P G P G L D P F P R H G G L A L Q P G P P G L H P F P F H P S L G P LE R E R L A L A A G P A L R PD M S YA E R L A A E R Q H A E R V A A L G N D P L A R L Q M L N V T P H H H Q H S H I H S H L H L H Q Q D A I H A A S A S V H P L I D P L A S G S H L T R I P Y P A G T L P N P L L P H P L H E N E V L R H Q L F A A P Y RD L P A S L S A P M S A A H Q L Q A M H A Q S A E L Q R L A L E Q Q Q W L H A H H P L H S V P L P A Q E D Y Y S H L K K E S D K P L _1190_

**a) COMPARISON OF ALL IDENTIFIED ATN1 CLONES**

Sequence comparison of the 16 ATN1 clones identified reveals a 378 residues common region that spans from residues 813-1190.

_813_ E E K E R E R E R E R E K E R E R E K E R E L E R S V K L A Q E G R A P V E C P S L G P V P H R P P F E P G S A V A T V P P Y L G P **D T P A L R T L S E Y A R P H V** M S P G N R N H P F Y V P L G A V D P G L L G Y N V P A L Y S S D P A A R E R E R E A R E R D L R D R L K P G F E V K P S E L E P L H G V P G P G L D P F P R H G G L A L Q P G P P G L H P F P F H P S L G P LE R E R L A L A A G P A L R P D M S YA E R L A A E R Q H A E R V A A L G N D P L A R L Q M L N V T P H H H Q H S H I H S H L H L H Q Q D A I H A A S A S V H P L I D P L A S G S H L T R I P Y P A G T L P N P L L P H P L H E N E V L R H Q L F A A P Y RD L P A S L S A P M S A A H Q L Q A M H A Q S A E L Q R L A L E Q Q Q W L H A H H P L H S V P L P A Q E D Y Y S H L K K E S D K P L _1190_

**(B) BCL11A Clones Obtained.** From several independent Y2H screens 4 clones of BCL11A have been identified using TLX-LBD (172-385) as a bait, and a total of 5 BCL11A using TLX-FL (1-385) as bait. The detailed sequence and description of these clones are indicated. The reported isoforms are produced by alternative splicing of 7 different exons (E1, E2, E-XS, E3, E4, E5-S and E5-L). The residue sequence belonging to E2, E3, and E4 is highlighted in blue, while the one belonging to E5-S is highlighted in orange and the one belonging to E5-L is highlighted in green. A novel isoform of BCL11A that we called isoform M, has been identified and corresponds to clone #1. The rest of the clones correspond only to isoform L.

**- BCL11A #1:**

Non-described isoform in the public protein sequence databases consulted.

Sequence deposited as BCL11A-M in GeneBank accession code JN852960.

Possibly novel Isoform: Composed of the C-terminal part of E4 (residues 451-744) from Isoform L + E5S (211-243).

**G T S D L V G S A S S A L K S V V A K F K S E N D P N L I P E N G D E E E E E D D E E E E E E E E E E E E E L T E S E R V D Y G F G L S L E A A R H H E N S S R G A V V G V G D E S R A L P D V M Q G M V L S S M Q H F S E A F H Q V L G E K H K R G H L A E A E G H R D T C D E D S V A G E S D R I D D G T V N G R G C S P G E S A S G G L S K K L L L G S P S S L S P F S K R I K L E K E F D L P P A A M P N T E N V Y S Q W L A G Y A A S R Q L K D P F L S F G D S R Q S P F A S S S E H S S E N G S L R F S T P P G E L D G G I S G R S G T G S G G S T P H I S G P G P G R P S S K E G R R S D T** **C L H T P P F G V V P R E L K M C G S F P D X A R E P L G S E K I**

**- BCL11A #2:**

Isoform L (AJ404612): C-terminal of E4 (residues 481-744) + E5L (residues 745-773).

**E N G D E E E E E D D E E E E E E E E E E E E E L T E S E R V D Y G F G L S L E A A R H H E N S S R G A V V G V G D E S R A L P D V M Q G M V L S S M Q H F S E A F H Q V L G E K H K R G H L A E A E G H R D T C D E D S V A G E S D R I D D G T V N G R G C S P G E S A S G G L S K K L L L G S P S S L S P F S K R I K L E K E F D L P P A A M P N T E N V Y S Q W L A G Y A A S R Q L K D P F L S F G D S R Q S P F A S S S E H S S E N G S L R F S T P P G E L D G G I S G R S G T G S G G S T P H I S G P G P G R P S S K E G R R S D T C S S H T P I R R S T Q R A Q D V W Q F S D G S S R A L K F**

**- BCL11A #3:**

Isoform L (AJ404612): Intronic Region + E2, E3 and E4 (residues 20-744) + E5L (residues 745-773).

**A G L W T P A R S R S S V Q R E E S S H P H N S E K V A L W K G A P G A P E S A E S G E G P R R R G E P W D R E G R E T R P A L P P F V P A R A E P L E A I L T D D E P D H G P L G A P E G D H D L L T C G Q C Q M N F P L G D I L I F I E H K R K Q C N G S L C L E K A V D K P P S P S P I E M K K A S N P V E V G I Q V T P E D D D C L S T S S R R I C P K Q E H I A D KL L H W R G L S S P R S A H G A L I P T P G M S A E Y A P Q G I C K D E P S S Y T C T T C K Q P F T S A W F L L Q H A Q N T H G L RI Y L E S E H G S P L T P R V G I P S G L G A E C P S Q P P L H G I H I A D N N P F N L L R I P G S V S R E A S G L A E G R F P P T P P L F S P P P R H H L D P H R I E R L G A E E M A L A T H H P S A F D R V L R L N P M A M E P P A M D F S R R L R E L A G N T S S P P L S P G R P S P M Q R L L Q P F Q P G S K P P F L A T P P L P P L Q S A P P P S Q P P V K S K S C E F C G K T F K F Q S N L V V H R R S H T G E K P Y K C N L C D H A C T Q A S K L K R H M K T H M H K S S P M T V K S D D G L S T A S S P E P G T S D L V G S A S S A L K S V V A K F K S E N D P N L I P E N G D E E E E E D D E E E E E E E E E E E E E L T E S E R V D Y G F G L S L E A A R H H E N S S R G A V V G V G D E S R A L P D V M Q G M V L S S M Q H F S E A F H Q V L G E K H K R G H L A E A E G H R D T C D E D S V A G E S D R I D D G T V N G R G C S P G E S A S G G L S K K L L L G S P S S L S P F S K R I K L E K E F D L P P A A M P N T E N V Y S Q W L A G Y A A S R Q L K D P F L S F G D S R Q S P F A S S S E H S S E N G S L R F S T P P G E L D G G I S G R S G T G S G G S T P H I S G P G P G R P S S K E G R R S D T** **C S S H T P I R R S T Q R A Q D V W Q F S D G S S R A L K F**

**- BCL11A #4:**

Isoform L (AJ404612): Intronic Region + E2, E3 and E4 (residues 20-744) + E5L (residues 745-773).

**A G L W T P A R S R S S V Q R E E S S H P H N S E K V A L W K G A P G A P E S A E S G E G P R R R G E P W D R E G R E T R P A L P P F V P A R A E P L E A I L T D D E P D H G P L G A P E G D H D L L T C G Q C Q M N F P L G D I L I F I E H K R K Q C N G S L C L E K A V D K P P S P S P I E M K K A S N P V E V G I Q V T P E D D D C L S T S S R R I C P K Q E H I A D KL L H W R G L S S P R S A H G A L I P T P G M S A E Y A P Q G I C K D E P S S Y T C T T C K Q P F T S A W F L L Q H A Q N T H G L RI Y L E S E H G S P L T P R V G I P S G L G A E C P S Q P P L H G I H I A D N N P F N L L R I P G S V S R E A S G L A E G R F P P T P P L F S P P P R H H L D P H R I E R L G A E E M A L A T H H P S A F D R V L R L N P M A M E P P A M D F S R R L R E L A G N T S S P P L S P G R P S P M Q R L L Q P F Q P G S K P P F L A T P P L P P L Q S A P P P S Q P P V K S K S C E F C G K T F K F Q S N L V V H R R S H T G E K P Y K C N L C D H A C T Q A S K L K R H M K T H M H K S S P M T V K S D D G L S T A S S P E P G T S D L V G S A S S A L K S V V A K F K S E N D P N L I P E N G D E E E E E D D E E E E E E E E E E E E E L T E S E R V D Y G F G L S L E A A R H H E N S S R G A V V G V G D E S R A L P D V M Q G M V L S S M Q H F S E A F H Q V L G E K H K R G H L A E A E G H R D T C D E D S V A G E S D R I D D G T V N G R G C S P G E S A S G G L S K K L L L G S P S S L S P F S K R I K L E K E F D L P P A A M P N T E N V Y S Q W L A G Y A A S R Q L K D P F L S F G D S R Q S P F A S S S E H S S E N G S L R F S T P P G E L D G G I S G R S G T G S G G S T P H I S G P G P G R P S S K E G R R S D T** **C S S H T P I R R S T Q R A Q D V W Q F S D G S S R A L K F**

**- BCL11A #5:**

Isoform L (AJ404612): C-terminal of E4 (residues 368-744) + E5L (residues 745-773).

**P P S Q P P V K S K S C E F C G K T F K F Q S N L V V H R R S H T G E K P Y K C N L C D H A C T Q A S K L K R H M K T H M H K S S P M T V K S D D G L S T A S S P E P G T S D L V G S A S S A L K S V V A K F K S E N D P N L I P E N G D E E E E E D D E E E E E E E E E E E E E L T E S E R V D Y G F G L S L E A A R H H E N S S R G A V V G V G D E S R A L P D V M Q G M V L S S M Q H F S E A F H Q V L G E K H K R G H L A E A E G H R D T C D E D S V A G E S D R I D D G T V N G R G C S P G E S A S G G L S K K L L L G S P S S L S P F S K R I K L E K E F D L P P A A M P N T E N V Y S Q W L A G Y A A S R Q L K D P F L S F G D S R Q S P F A S S S E H S S E N G S L R F S T P P G E L D G G I S G R S G T G S G G S T P H I S G P G P G R P S S K E G R R S D T** **C S S H T P I R R S T Q R A Q D V W Q F S D G S S R A L K F**

**- BCL11A #6:**

Isoform L (AJ404612): Unidentified region + E2, E3 and E4 (residues 20-744) + E5L (residues 745-773).

**G I R R G A R R C E V T A R P A E P L E A I L T D D E P D H G P L G A P E G D H D L L T C G Q C Q M N F P L G D I L I F I E H K R K Q C N G S L C L E K A V D K P P S P S P I E M K K A S N P V E V G I Q V T P E D D D C L S T S S R R I C P K Q E H I A D KL L H W R G L S S P R S A H G A L I P T P G M S A E Y A P Q G I C K D E P S S Y T C T T C K Q P F T S A W F L L Q H A Q N T H G L RI Y L E S E H G S P L T P R V G I P S G L G A E C P S Q P P L H G I H I A D N N P F N L L R I P G S V S R E A S G L A E G R F P P T P P L F S P P P R H H L D P H R I E R L G A E E M A L A T H H P S A F D R V L R L N P M A M E P P A M D F S R R L R E L A G N T S S P P L S P G R P S P M Q R L L Q P F Q P G S K P P F L A T P P L P P L Q S A P P P S Q P P V K S K S C E F C G K T F K F Q S N L V V H R R S H T G E K P Y K C N L C D H A C T Q A S K L K R H M K T H M H K S S P M T V K S D D G L S T A S S P E P G T S D L V G S A S S A L K S V V A K F K S E N D P N L I P E N G D E E E E E D D E E E E E E E E E E E E E L T E S E R V D Y G F G L S L E A A R H H E N S S R G A V V G V G D E S R A L P D V M Q G M V L S S M Q H F S E A F H Q V L G E K H K R G H L A E A E G H R D T C D E D S V A G E S D R I D D G T V N G R G C S P G E S A S G G L S K K L L L G S P S S L S P F S K R I K L E K E F D L P P A A M P N T E N V Y S Q W L A G Y A A S R Q L K D P F L S F G D S R Q S P F A S S S E H S S E N G S L R F S T P P G E L D G G I S G R S G T G S G G S T P H I S G P G P G R P S S K E G R R S D T** **C S S H T P I R R S T Q R A Q D V W Q F S D G S S R A L K F**

**- BCL11A #7:**

Isoform L (AJ404612): C-terminal of E4 (residues 586-744) + complete E5L (residues 745-773).

**D E D S V A G E S D R I D D G T V N G R G C S P G E S A S G G L S K K L L L G S P S S L S P F S K R I K L E K E F D L P P A A M P N T E N V Y S Q W L A G Y A A S R Q L K D P F L S F G D S R Q S P F A S S S E H S S E N G S L R F S T P P G E L D G G I S G R S G T G S G G S T P H I S G P G P G R P S S K E G R R S D T C** **S S H T P I R R S T Q R A Q D V W Q F S D G S S R A L K F**

**- BCL11A #8:**

Isoform L (AJ404612): C-terminal of E4 (residues 380-744) + complete E5L (residues 745-773).

**E F C G K T F K F Q S N L V V H R R S H T G E K P Y K C N L C D H A C T Q A S K L K R H M K T H M H K S S P M T V K S D D G L S T A S S P E P G T S D L V G S A S S A L K S V V A K F K S E N D P N L I P E N G D E E E E E D D E E E E E E E E E E E E E L T E S E R V D Y G F G L S L E A A R H H E N S S R G A V V G V G D E S R A L P D V M Q G M V L S S M Q H F S E A F H Q V L G E K H K R G H L A E A E G H R D T C D E D S V A G E S D R I D D G T V N G R G C S P G E S A S G G L S K K L L L G S P S S L S P F S K R I K L E K E F D L P P A A M P N T E N V Y S Q W L A G Y A A S R Q L K D P F L S F G D S R Q S P F A S S S E H S S E N G S L R F S T P P G E L D G G I S G R S G T G S G G S T P H I S G P G P G R P S S K E G R R S D T C** **S S H T P I R R S T Q R A Q D V W Q F S D G S S R A L K F**

**- BCL11A #9:**

Isoform L (AJ404612): C-terminal of E4 (residues 481-744) + E5L (residues 745-773).

**E N G D E E E E E D D E E E E E E E E E E E E E L T E S E R V D Y G F G L S L E A A R H H E N S S R G A V V G V G D E S R A L P D V M Q G M V L S S M Q H F S E A F H Q V L G E K H K R G H L A E A E G H R D T C D E D S V A G E S D R I D D G T V N G R G C S P G E S A S G G L S K K L L L G S P S S L S P F S K R I K L E K E F D L P P A A M P N T E N V Y S Q W L A G Y A A S R Q L K D P F L S F G D S R Q S P F A S S S E H S S E N G S L R F S T P P G E L D G G I S G R S G T G S G G S T P H I S G P G P G R P S S K E G R R S D T C S S H T P I R R S T Q R A Q D V W Q F S D G S S R A L K F**

**a) COMPARISON OF ALL IDENTIFIED BCL11A CLONES**

Detailed sequence analysis of all BCL11a clones identified revealed a common region that spans residues 586-744:

_586_**DEDSVAGESDRIDDGTVNGRGCSPGESASGGLSKKLLLGSPSSLSPFSKRIKLEKEFDLPPAAMPNTENVYSQWLAGYAASRQLKDPFLSFGDSRQSPFASSSEHSSENGSLRFSTPPGELDGGISGRSGTGSGGSTPHISGPGPGRPSSKEGRRSDTC**_744_
